# Pulmonary emphysema: the assessment of lung perfusion with Dual-Energy CT and pulmonary scintigraphy

**DOI:** 10.1007/s11547-024-01883-y

**Published:** 2024-09-10

**Authors:** Alessandra Borgheresi, Elisa Cesari, Andrea Agostini, Myriam Badaloni, Sofia Balducci, Elisabetta Tola, Valeria Consoli, Andrea Palucci, Luca Burroni, Marina Carotti, Andrea Giovagnoni

**Affiliations:** 1 Department of Clinical, Special and Dental Sciences, University Politecnica Delle Marche, Via Tronto 10/A, 60126 Ancona, AN Italy; 2 Department of Radiological Sciences, Division of Clinical Radiology, University Hospital “Azienda Ospedaliero Universitaria Delle Marche”, Via Conca 71, 60126 Ancona, AN Italy; 3 School of Radiology, University Politecnica Delle Marche, Via Tronto 10/A, 60126 Ancona, AN Italy; 4 Department of Radiological Sciences. Division of Nuclear Medicine, University Hospital “Azienda Ospedaliero Universitaria Delle Marche”, Via Conca 71, 60126 Ancona, AN Italy

**Keywords:** Dual energy CT, Spectral imaging, Lung, COPD, Emphysema, Scintigraphy

## Abstract

**Aim:**

To assess the correlation of quantitative data of pulmonary Perfused Blood Volume (PBV) on Dual-Energy CT (DECT) datasets in patients with moderate – severe Pulmonary Emphysema (PE) with Lung Perfusion Scintigraphy (LPS) as the reference standard. The secondary endpoints are the correlation between the CT densitometric analysis and the visual assessment of parenchymal destruction with PBV.

**Materials and Methods:**

Patients with moderate – severe PE candidate to Lung Volumetric Reduction (LVR), with available a pre-procedural LS and a contrast-enhanced DECT were retrospectively included. DECT studies were performed with a 3rd generation Dual-Source CT and the PBV was obtained with a 3-material decomposition algorithm. The CT densitometric analysis was performed with a dedicated commercial software (Pulmo3D). The Goddard Score was used for visual assessment. The perfusion LS were performed after the administration of albumin macroaggregates labeled with ^99m^Technetium. The image revision was performed by two radiologists or nuclear medicine physicians blinded, respectively, to LS and DECT data. The statistical analysis was performed with nonparametric tests.

**Results:**

Thirty-one patients (18 males, median age 69 y.o., interquartile range 62–71 y.o.) with moderate – severe PE (Median Goddard Score 14/20 and 31% of emphysematous parenchyma at quantitative CT) candidate to LVR were retrospectively included. The median enhancement on PBV was 17 HU. Significant correlation coefficients were demonstrated between lung PBV and LS, poor in apical regions (Rho = 0.1–0.2) and fair (Rho = 0.3–0.5) in middle and lower regions. No significant correlations were recorded between the CT densitometric analysis, the visual score, and the PBV.

**Conclusions:**

Lung perfusion with PBV on DECT is feasible in patients with moderate – severe PE candidate to LVR, and has a poor to fair agreement with LPS.

## Introduction

Pulmonary emphysema (PE) is defined as the abnormal permanent enlargement of airways distal to the terminal bronchiole with alveolar destruction, without any fibrosis, and it is included in chronic obstructive pulmonary disease (COPD) [1, 2]. PE is characterized by obstructive ventilatory defects, reduced diffusion capacity, and parenchymal destruction is also often associated with altered alveolar ventilation/perfusion ratio (V/Q) [3, 4]. Ventilatory and Perfusion functional parameters assessed at scintigraphy are altered when at least one-third of the lung parenchyma is involved, making them poorly sensitive [4, 5]. Moreover, alveolar destruction and hypoxic vasoconstriction reduce regional perfusion in the emphysematous lung [4]. However, the distribution of perfusion damage does not necessarily correlate with the area of parenchymal destruction [4, 6–9].

Patients with advanced PE, poorly controlled with medical treatment, may take benefit from endoscopic or surgical Lung Volume Reduction (LVR) [10, 11]. These procedures aim to reduce the size disproportion between the hyperinflate pulmonary parenchyma and the volume of the chest cavity, thus restoring the forces acting on the elastic recoil of the bronchiolar walls [12]. As consequences, these procedures lead to an improved bronchiolar airflow with a global beneficial effect on the cardiothoracic function and on physical exercise [12–14]. The early diagnosis, classification, and assessment of disease extension strongly relies on High-Resolution Computed Tomography (HRCT) [15–17]. However, the management of candidates for LVR must include the assessment of lung perfusion, since perfused parenchyma should be preserved, while the pulmonary portions with impaired perfusion need to be treated [6, 7, 18, 19].

The assessment of PE on HRCT may be performed with visual, semiquantitative scores (Visual Score, VS), or with automatic or semiautomatic software that quantifies the percentage of pathological parenchyma in each volume using densitometric thresholds and morphological changes [18, 20–25]. On the one side, VS usually has an easy application but is time-consuming and has a low intra- and inter-rater agreement [18, 20–23]. Conversely, semi-automatic software has the potential for a more objective quantification with lower variability [18, 24].

Several methods are available for the assessment of lung perfusion; among them, the Lung Perfusion Scintigraphy (LPS) is one of the most widely used [25]. LPS provides a two-dimensional compression of a volumetric distribution of a radiotracer and does not provide morphological information [6, 26]. Dual-Energy CT (DECT) with material decomposition allows for effective iodine quantification [27]. The application of a specific three-material decomposition algorithm for the lung parenchyma provides the Perfused Blood Volume (PBV), namely the perfused blood volume into a unit mass of lung tissue, which is translated to a whole-lung map to assess the parenchymal perfusion [28, 29]. The agreement of PBV and nuclear medicine studies, mostly with qualitative methods, has been more extensively evaluated in pulmonary embolism [29–32], while little evidence is available for PE [33].

This study aims to correlate the quantitative PBV with LPS parameters in patients with moderate to severe PE candidate to endoscopic LVR. Secondary endpoints are the correlation between the lung PBV and the densitometric analysis with the semiautomatic software, and the correlation between perfusion abnormalities at PBV with parenchymal destruction at VS.

## Materials and methods

### Patient selection

This study was conducted in accordance with the Declaration of Helsinki. In agreement with our local IRB, due to the data being registered anonymously and the study’s retrospective design, formal ethical approval by the IRB was not required.

Patients with moderate-severe PE, in evaluation for endoscopic LVR, with pulmonary angio-DECT imaging between February 2018 and March 2022, who underwent planar LPS within 15 days of the DECT at our Institution (Azienda Ospedaliero Universitaria delle Marche) were included. Patients with imaging of inadequate quality (i.e., pulmonary artery enhancement < 200 HU, motion artifacts, underlying pulmonary diseases such as consolidations or pulmonary embolism), or with incomplete imaging data (DECT or LPS data not available) were not included.

### DECT acquisition protocol

CT examinations were performed using 3rd generation Dual Source DECT (Somatom Force, Siemens Healthineers). The basal acquisition was performed in deep inspiration and reconstructed with a medium and sharp kernel (120 kV, modulated mA, model-based iterative reconstruction, ADMIRE, strength 3, kernels Br40 and Bl64) with a slice thickness and spacing of 1 mm/0.7 mm [34, 35]. The study was completed with a DECT acquisition (90/150Sn, modulated mA, ADMIRE 3, reconstruction kernels Br40, BL64, and Qr40, thickness/spacing: 1/0.7 mm) after the administration of intravenous contrast material (Iopamidol 370 mgI/mL, Iopamiro 370, Bracco; 1 mL/kg, 3,5 – 5 ml/s); the acquisition time was optimized with bolus tracking technique (Threshold: 100 HU in pulmonary trunk; acquisition delay: 6 s).

### CT visual analysis

Visual quantification of emphysema was performed with Goddard VS on HRCT basal images by two readers in consensus and blinded to LS (AA, AB, with 15 and 10 years of experience in thoracic imaging), after the quality control of the DECT study (i.e., adequate enhancement of the pulmonary artery) and exclusion of concomitant pulmonary diseases [20]. Both lungs were evaluated at three levels: upper, middle, and lower. The apical zone was examined at an axial scan passing 1 cm above the upper margin of the aortic arch; the middle lung was examined at an axial scan passing 1 cm below the tracheal carina; the basal lung was examined 3 cm above the diaphragmatic dome. A percentage score of emphysematous parenchyma was assigned at these zones, bilaterally. In particular, no evidence of emphysema: score 0; emphysematous areas less than 25%: score 1; between 26 and 50%: score 2; between 51 and 75%: score 3; greater than 75%: score 4. These scores were summed, and the total value was the severity score of PE. Conventionally, the three regions of the lung considered for the visual analysis, and for the further segmentation (see below), were labeled as left (L1, L2, L3) and right (R1, R2, R3) in cranio-caudal direction.

### Densitometric analysis with semi-automatic software

Basal images reconstructed with medium kernel (Br40) were processed with semiautomatic software for densitometric analysis (Pulmo3D on Syngo.via, Siemens Healthineers) by the same radiologists blinded to LS [34, 35]. The software quantifies the percentage of voxels with attenuation values lower than the threshold of -950 HU for the entire pulmonary parenchyma, the right and left lungs separately and the lower, middle and upper zones of equal volume (Volume Based, VB) of each lung separately (R1-3; L1-3). The Mean Lung Densities (MLD) were also recorded for each patient.

### Calculation of PBV

The two radiologists in consensus and blinded to LS processed the post-contrast DECT datasets with dedicated three-material decomposition on DE Lung Analysis (Syngo.via, Siemens Healthineers) to calculate the lung PBV. Iodine distribution and PBV maps were rendered on axial, sagittal, and coronal planes and in 3D Volume Rendering (VR). The measurements were performed after normalization of iodine distribution on the Pulmonary artery enhancement: a standardized region of interest (ROI) sized 0.5 cm2 was placed in the pulmonary trunk on axial images. Lung partitioning was performed automatically using the Distance Based (DB) mode. The lungs were divided by two horizontal lines into three fields of equivalent cranio-caudal length: upper, middle, and lower (R1-3; L1-3). DB is a mode of lung segmentation more similar to that used in scintigraphy than the technique of VB segmentation used in densitometric analysis.

### LPS acquisition, post-processing, and assessment

Perfusion Scintigraphy examinations were performed with γ-chamber Infinia (GE Healthcare). Eight standard planar projections (anterior, posterior, right lateral, left lateral, right posterior oblique, left posterior oblique, right anterior oblique, left anterior oblique) were acquired after intravenous administration of albumin macroaggregates labeled with ^99m^Technetium (^99m^Tc-MAA) [4]. The post-processing and image revision was performed with dedicated software by two nuclear medicine physicians in consensus and blinded to the DECT datasets (LB, AP, 30 and 10 years of experience respectively): each anterior and posterior digital planar scintigraphy projection of each lung was manually demarcated within a rectangular ROI which is semi-manually subdivided into three regions (upper, middle, lower) of approximately equivalent areas by two horizontal lines (R1-3; L1-3). The anteroposterior geometric mean of the detected counts was automatically calculated for all areas corresponding to anterior and posterior projections of each lung. The estimated lung perfusion was obtained in absolute k-count value for each lung field examined [26].

## Statistical analysis

Statistical analysis of the data was performed on MedCalc Statistic Software (v20.211, MedCalc Software). All the quantitative variables were tested for normality (D’Agostino-Pearson test), and after the rejection of normal distribution, the variables were expressed as median values and interquartile ranges, and nonparametric tests were used. In particular, Wilcoxon or Friedman Tests were used when appropriate to assess differences among related samples, and correlation between DECT and LPS was evaluated with Rank Correlation (Spearman’s Rho), the values were interpreted, in absolute values, as perfect (1), very strong (0.8–0.9), moderate (0.6–0.7), fair (0.3–0.5), poor (0.1–0.2), and none (0) [36]. P values < 0.05 were considered significant.

## Results

Fifty-five patients underwent to contrast-enhanced DECT study as treatment planning of LVR. Out of them, 5 had DECT studies of inadequate quality (3 patients with motion artifacts; 2 patients with inadequate enhancement of the pulmonary artery), and 19 had incomplete or inadequate imaging data (scintigraphy data not available). The final population included 31 patients with moderate-to-severe PE candidate to LVS. The median age was 69 years and 18 candidates were male, with a median Goddard VS of 14/24 (interquartile range 8–20).

Quantification of PE evaluated by Pulmo3D application of Syngo.via software had a median value of 31%, with a median value of mean lung density (MLD) of -903 HU. The median PBV value was obtained as an absolute value of 16.5 HU (Table 1).

**Table 1:** Patient population and main findings

Study population	
Patients	31
Age (y.o.)	69
Median, IQR	(62–71)
Sex (M/F)	18/13
Goddard VS Median, IQR	14 (8–20)
Percentage of emphysematous parenchyma (%, Densitometric analysis, semi-automatic software) Median, IQR	31% (27%–33%)
MLD (HU, densitometric analysis, semi-automatic software) Median, IQR	−903 (−909 to –896)
PBV value (HU, both lungs) Median, IQR	17 (13–19)
Lung Perfusion Scintigraphy (kct) Median, IQR	689 (83–701)

*VS* Visual score, *IQR* interquartile range, *MLD* mean lung density, *PBV* perfused blood volume, *kct* kilo-counts.

Table 2 reports the semi—quantitative and quantitative findings for each lung region. Significant differences were recorded among median Goddard visual scores, CT densitometric parameters, and LS of the different regions (Friedman *p* < 0.05). No significant differences were recorded for PBV parameters across the different lung regions.

**Table 2:** Quantitative and semi-quantitative findings for each lung zone

	Whole Lungs			Left Lung				Right Lung			
	Left Lung	Right Lung	Wilcoxon P^§^	L1	L2	L3	Friedman P*	Rl	R2	R3	Friedman P*
Goddard VS Median (IQR)	7.5 (4–10)	7.0 (3.5–10)	0.7290	3 (1–4)	2.5 (1–4)	2 (1–3)	**0.02060**	3.5 (1–4)	2 (1–3.5)	2 (1–2.5)	**0.00290**
PE (CT Densitometry). % Median (IQR)	29.9 (27.0–33.0)	31 (26.3–33.2)	0.6394	33.7 (29.9–38.3)	31.0 (27.4–33.2)	24.8 (22.3–29.8)	**< 0.00001**	34.0 (24.5- 37.7)	30.6 (25.6- 33.2)	26.9 (25.0−30.9)	0.08150
MLD (CT Densitometry). % Median (IQR)	−902 (−911 to −894)	−904 (−911 to −887)	0.6374	−919 (−935 to −907)	−900 (−911 to −890)	−884 (−902 to −868)	**< 0.00001**	−921 (−933 to − −889)	-900 (−907 to − −886)	−889 (−905 to −873)	**0.00008**
PBV (HU). Median (IQR)	16 (12–19)	17.5 (12–19)	0.0802	14 (10.5–19.5)	15 (12–27)	15.5 (12.5–26)	0.59533	16 (12.5–19)	16.5 (13–19)	16 (12–19)	0.13808
LPS (kct). Median (IQR)	261 (155–330)	329 (220–437)	0.0516	47 (41–62)	130 (74−175)	55 (46−92)	** < 0.00001**	56 (38−71)	156 (117–242)	87 (55–125)	**<0.00001**

*VS* Visual score, *PE* pulmonary Emphysema, *PBV* perfused blood volume, *LPS* perfusion lung scintigraphy, *IQR* interquartile range, *MLD* mean lung density. L1, L2, L3, R1, R2, R3: craniocaudal regions of the Left and Right Lungs

§Wilcoxon test significance

*Friedman test significance. Significant *p* values are in **bold**

**Fig. 1:**
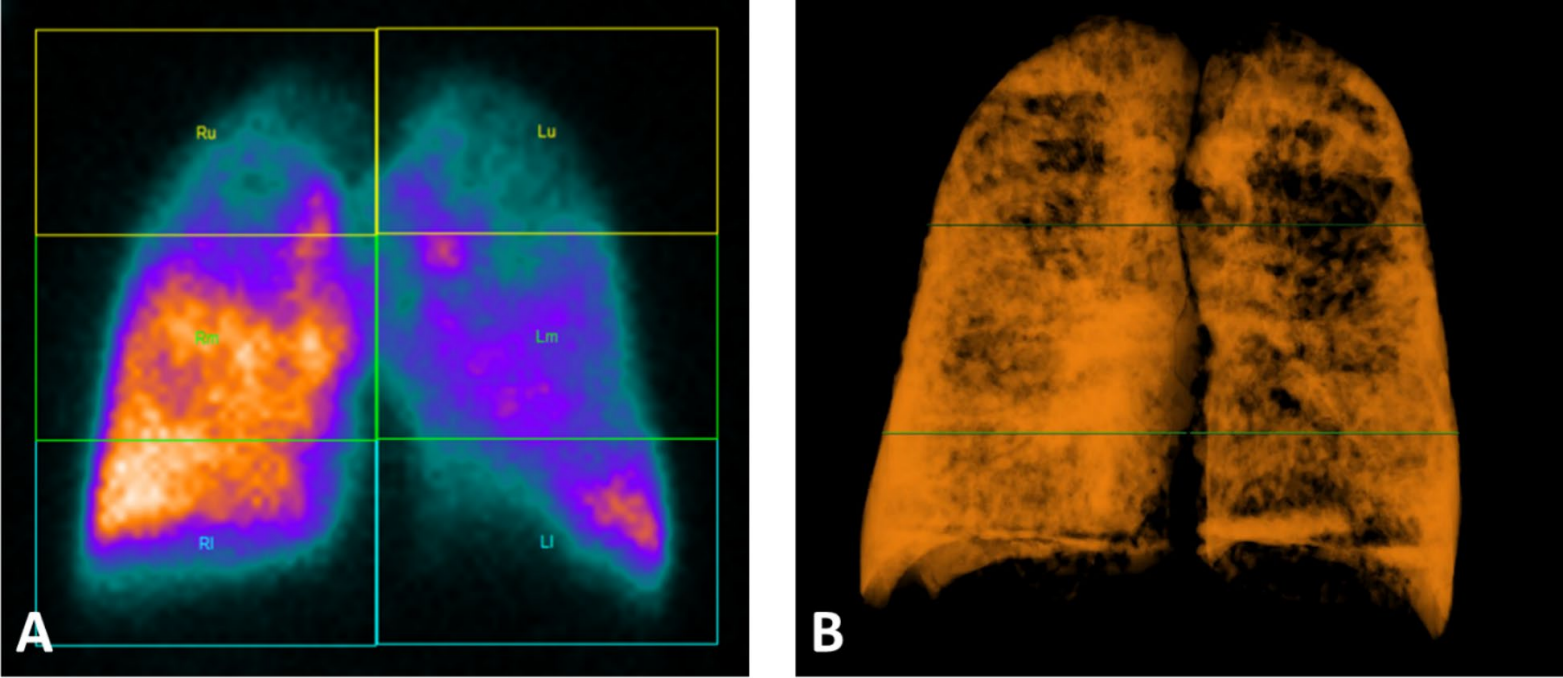
Correlation between LPS (a) and PBV (b). Visually, there is a positive correlation in each lung zone (apical, middle and lower regions)

**Fig. 2:**
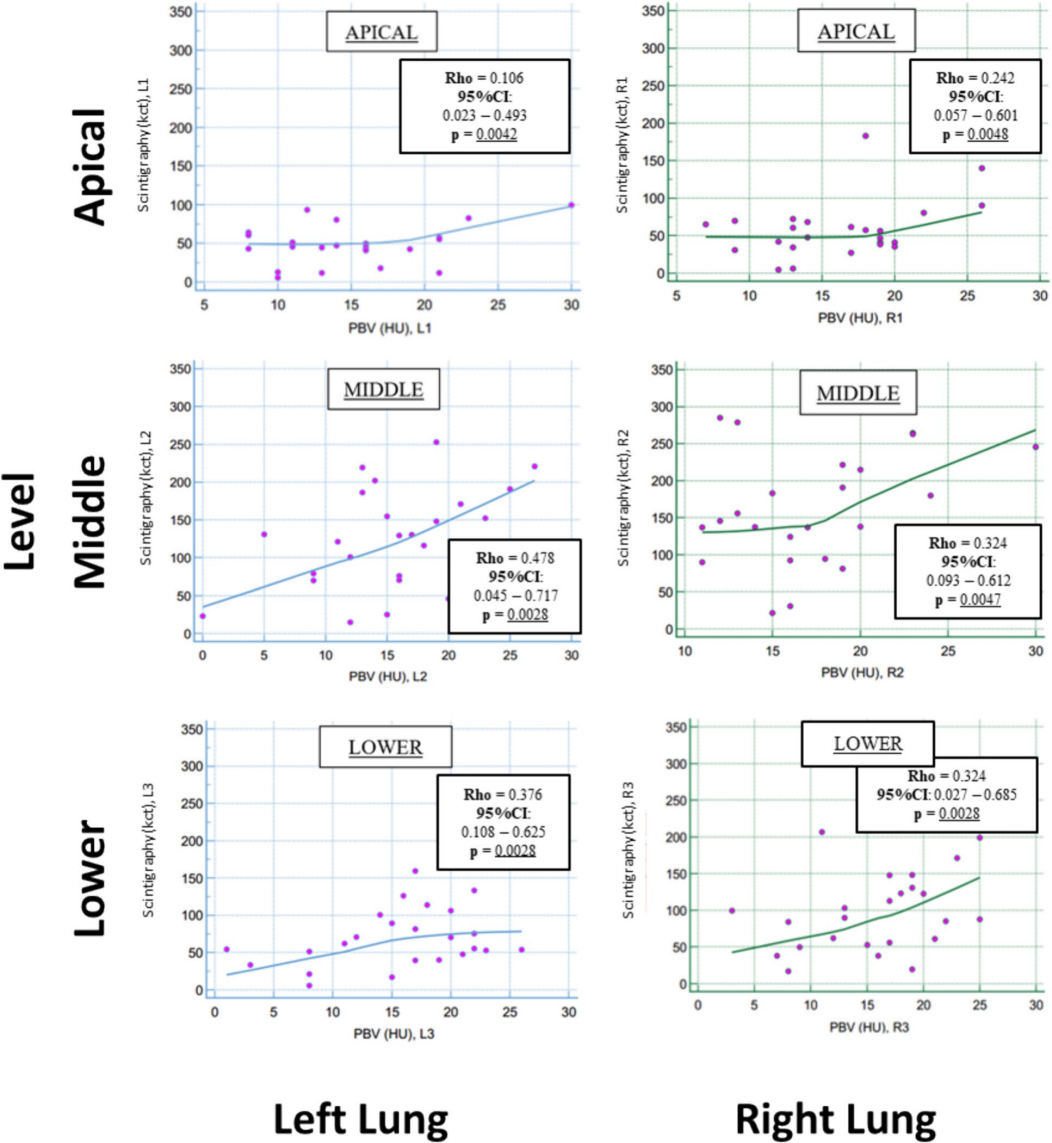
Rank correlation (Spearman Rho) between PBV (HU) and lung Scintigraphy (kilo-count, kct). The figure reports the Spearman’s Rho coefficients, the relative 95% confidence intervals (95%CI) and the p-values. Significant correlations were found for the two methods for each region of the right (R1, R2, R3) and left lung (L1, L2, L3) regions. Significant p-values are underlined

Regional PBV in absolute value (HU) in DB mode and perfusion values obtained by LPS in k-counts were correlated (Figs.1, 2). More in detail, a significant correlation was found between PBV values and k-count for each region of the right and left lungs (Spearman Rho ranging between 0.106 and 0.478).

**Fig. 3:**
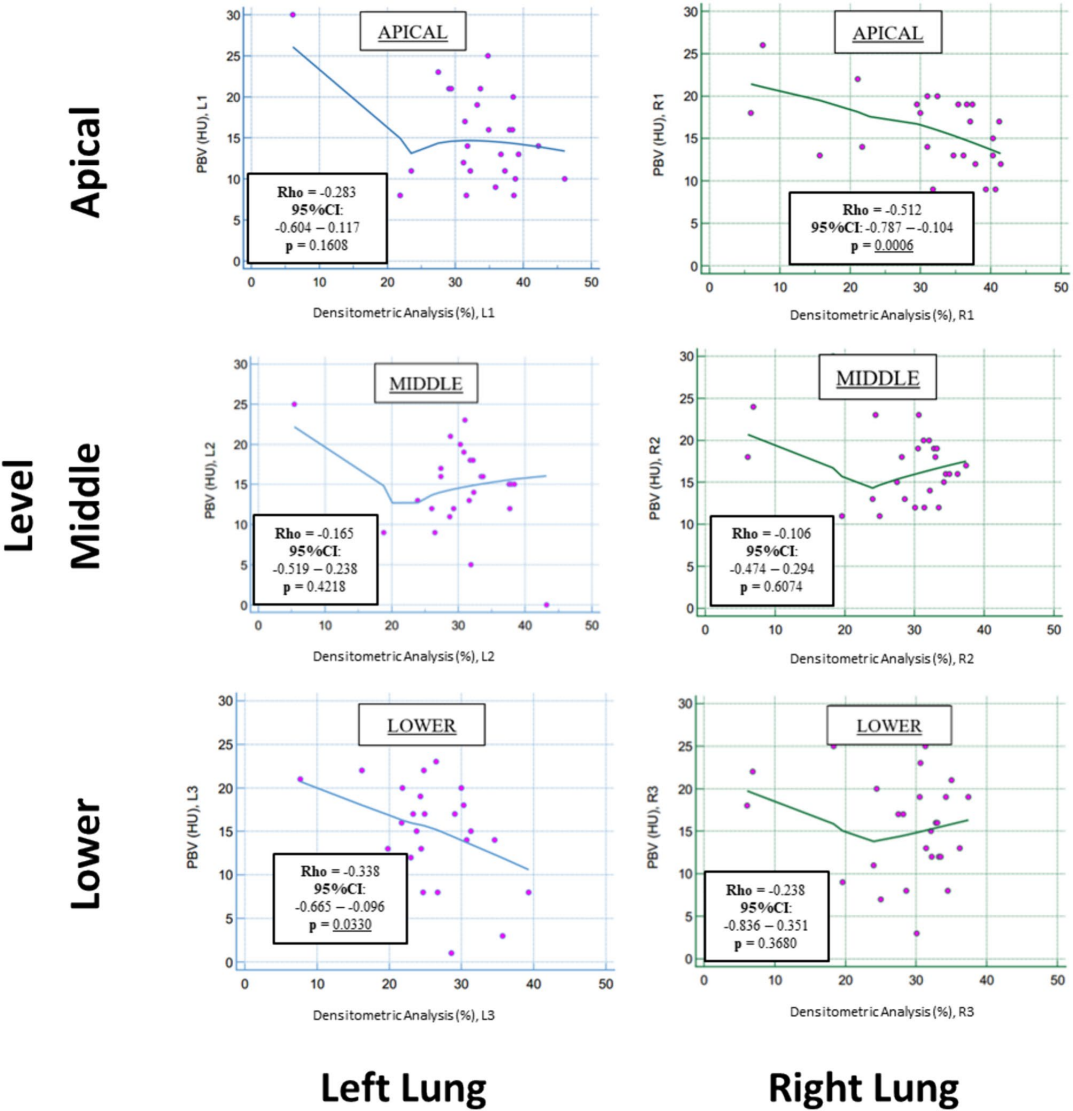
Rank correlation (Spearman Rho) between PBV on DECT (HU) and densitometric analysis with semi-automatic software (pulmonary emphysema, PE, % value). The figure reports the Spearman’s Rho coefficients, the relative 95% confidence intervals (95%CI) and the p-values for each pulmonary region (L1, L2, L3, R1, R2, R3). A negative trend for correlation between the PBV and PE% was recorded, though significant only for right apical and left lower regions. Significant p-values are underlined

Figure 3 reports the rank correlation (Spearman Rho) between the densitometric analysis with semi-automatic software (Pulmo 3D on Syngo.via) and lung PBV. In this case, a negative correlation trend was found between the extension of parenchymal destruction and perfusion; however, significant values were found only in the upper right region and lower left region.

**Fig. 4:**
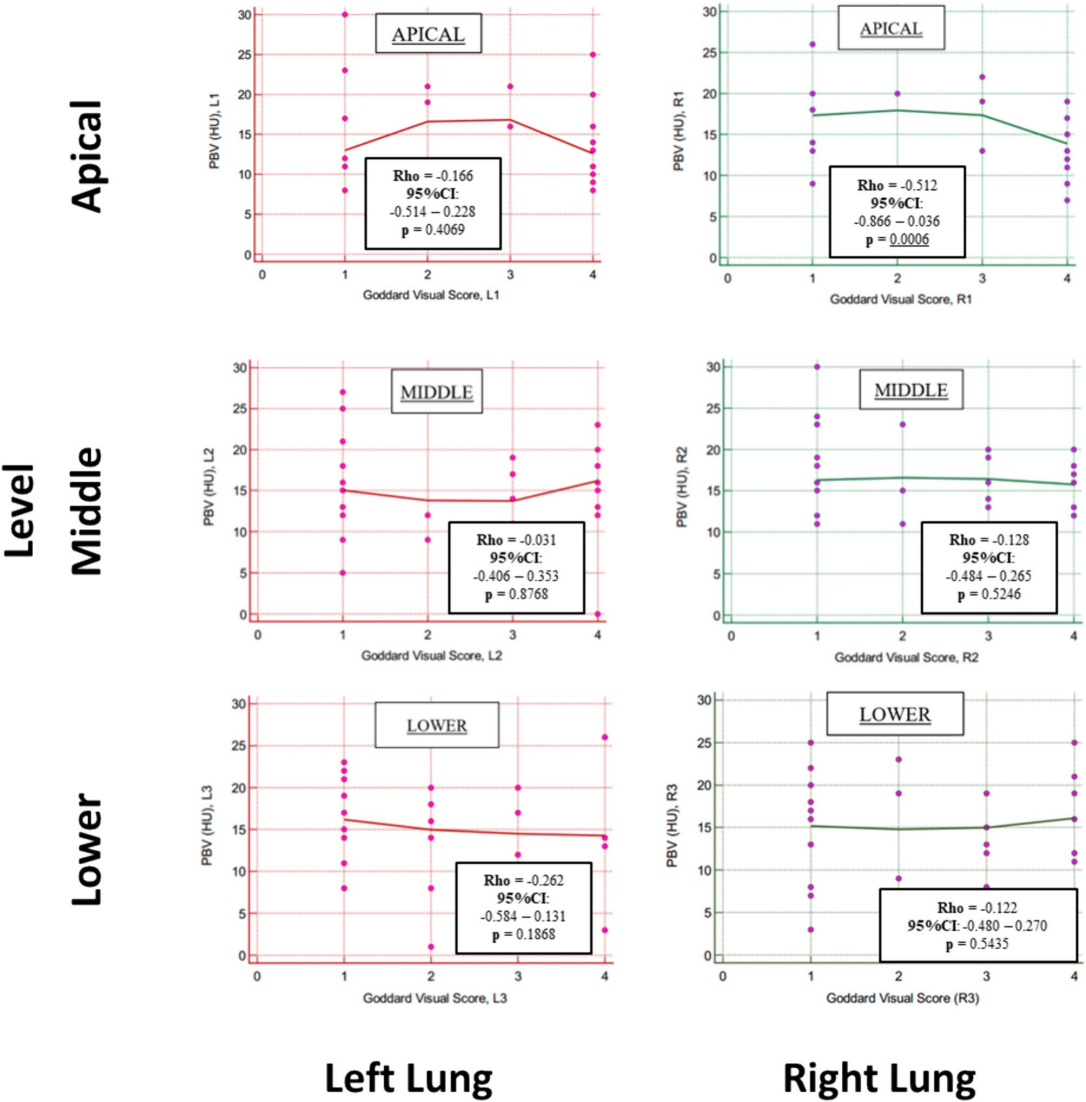
Rank correlation (Spearman Rho) between PBV on DECT (HU) and Goddard visual score (VS). The figure reports the Spearman’s Rho coefficients, the relative 95% confidence intervals (95%CI) and the p-values for each pulmonary region (L1, L2, L3, R1, R2, R3). Significant correlations were found only for the apical region of the right lung. Significant p-values are underlined

This statistical trend was confirmed by the correlation analysis between the Goddard VS and lung PBV (Fig. 4), where a significant, negative correlation was found only in the upper region of the right lung.

## Discussion

This study aims to compare the quantitative assessment if lung perfusion on DECT (the lung PBV) with quantitative data of LPS: we found statistically significant, poor to fair correlation between the two techniques, with slightly higher correlation coefficients in middle and basal regions of right and left lungs (Fig. 2). Regarding the secondary endpoint, the correlation between pulmonary perfusion assessed with lung PBV and parenchymal destruction by PE, assessed with the Goddard semiquantitative score and with a dedicated, semi-automatic software, were not significant with few exceptions in upper or lower pulmonary regions (Figs. 3, 4).

These results deserve some considerations. In our study, both the software for the post-processing of DECT and scintigraphy data divided the lungs in three regions: fair, significant concordance was demonstrated for the middle and lower regions, while only a poor concordance was recorded in the apical regions. This finding can be explained by accounting several aspects. First, while the Lung PBV for DECT analysis uses an automatic method for the division in cranio-caudal equally distant regions (DB mode), the same method is semi-automatic for LPS, introducing a variability source. Second, the calculation of PBV use a three-material decomposition, where the spectral curves of air and lung are included for the calculation of the distribution of iodine. It has been demonstrated that the accuracy of iodine quantification can be influenced by variation of the effective atomic number and electron density given by pathological conditions (i.e., pulmonary consolidations) [28]. It could be hypothesized that different grades of parenchymal destruction in PE may slightly but significantly affect the estimation of PBV in PE. Moreover, the post processing of DECT datasets is prone to beam-hardening artifacts, which can be more significant in regions close to the superior vena cava and in paracardiac parenchyma [37].

While the radionuclide perfusion and ventilation examinations have an established role in the management of COPD and PE, the iodine spectral analysis on DECT is still under investigation [26, 33, 38]. On the one side, perfusion scintigraphy is based on the intravenous administration of ^99m^Technetium—labeled albumin macroaggregates (^99m^TcMAA) which causes a microembolization of the pulmonary circulation resolving in 6–8 h, and the γ-rays emitted are detected by the γ-chamber [26]. On the other side, the assessment of pulmonary perfusion with DECT is derived from the iodine density map during the first pass of the contrast material in the pulmonary microcirculation [28]. The specific software for PBV is based on three-material decomposition to produce material-specific images from air, soft tissue, and iodine. After thresholding for the selection of the pulmonary parenchyma, the iodine density is superimposed to the virtual non-contrast images [39, 40]. Several studies assessed the diagnostic performance of Lung perfusion with DECT, particularly in patients with pulmonary thromboembolism (PTE). Sueyoshi et al. demonstrated significant different lung perfusion in patients with and without PTE [41]. These results were confirmed by Sakamoto et al. in patients with acute PTE of different grades of severity [39]. Dournes et al. compared the diagnostic performance of DECT with lung scintigraphy in patients with chronic thromboembolic pulmonary hypertension demonstrating a fair agreement (k = 0.44) between the two methods [30]. Renapurkar et al. performed the comparison between DECT and SPECT on a similar population of patients with chronic pulmonary hypertension, recording a lower inter-method agreement of k = 0.22–0.25 [42]. Regarding pulmonary emphysema, Gietema et al. compared the lung perfusion from DECT with scintigraphy, finding a good correlation between the two techniques [33]. These results were confirmed by Si-Mohamed et al. comparing the lung perfusion on DCT and SPECT/CT in PE [43]. However, the assessment of parenchymal involvement at HRCT nor the correlation with PBV were not performed in both studies [33, 43].

Even though the treatment planning of LVR mainly relies on the HRCT assessment of emphysema, the LPS still have a pivotal role by highlighting eventual mismatch of ventilation and perfusion in PE. It must be highlighted that lung PBV is a volumetric technique, while scintigraphy provides planar information [26]. This aspect is of clinical relevance, since the PBV on DECT provides more detailed spatial information than LPS. Moreover, the perfusion data of PBV can be superimposed, pixel by pixel, to the morphological information provided by the HRCT, thus giving the potential to DECT of being a one-stop-shop examination for the treatment planning of LVR in PE [26, 33].

Our data show a lack of concordance between the degree of parenchymal destruction and the hypoperfusion of the relative areas, confirming the previous studies [21]. However, it must be highlighted that also in this case the different segmentation techniques (volume-based for densitometric analysis and DB for the PBV) can be a source of variability of results. This trend was confirmed when the parenchyma destruction with VS was compared with the regional, PBV data, confirming the additional information provided by the perfusion assessment in addition to parenchymal destruction [6–8, 18, 19, 21].

The present study has several limitations. It is a retrospective, single-center study on a small cohort of patients with moderate-severe PE assessed with one DECT platform: the reproducibility of results should be assessed on different DECT platforms and in patients with different disease severity.

In conclusion, the calculation of PBV demonstrated a moderate correlation with scintigraphy data, with no significant correlation with the degree of parenchymal destruction assessed with semiautomatic software and VS in patients with moderate – severe PE candidate do LVR.
